# Worse and worse off: the impact of lymphedema on work and career after breast cancer

**DOI:** 10.1186/s40064-016-2300-8

**Published:** 2016-05-17

**Authors:** John Boyages, Senia Kalfa, Ying Xu, Louise Koelmeyer, Helen Mackie, Hector Viveros, Lucy Taksa, Paul Gollan

**Affiliations:** Department of Clinical Medicine, Faculty of Medicine and Health Sciences, 2 Technology Place, Macquarie University, Sydney, NSW 2109 Australia; Department of Marketing and Management, Faculty of Business and Economics, Macquarie University, Sydney, NSW 2109 Australia; Macquarie University Hospital, Macquarie University, Sydney, NSW Australia; Mount Wilga Private Hospital, 66 Rosamond Street, Hornsby, NSW 2077 Australia; Australian Institute for Business and Economics, Faculty of Business, Economics and Law, University of Queensland, Brisbane, QLD Australia

## Abstract

**Purpose:**

Our study examines the impact of breast cancer-related lymphedema on women’s work and career. Our research addresses a significant knowledge gap regarding the additional impact of lymphedema on breast cancer survivors.

**Methods:**

An online national survey was conducted with 361 women who either had breast cancer without lymphedema (Group 1, n = 209) or breast cancer with lymphedema (Group 2, n = 152). Participant recruitment was supported by the Breast Cancer Network Australia and the Australasian Lymphology Association.

**Results:**

Both breast cancer and lymphedema had a significant negative influence on women’s work and career. Respondents reported changes in employment resulting from stress and/or physical impairment, which affected attendance and work performance. The perceived negative impact of breast cancer on respondents’ work and career was noticeably greater in Group 2 (63 %) than Group 1 (51 %) (p = 0.03). Of the participants who were in paid employment at some time (either at diagnosis of lymphedema or at the time of the survey (n = 103), 43 (42 %) indicated that lymphedema impacted their work performance. The impact of lymphedema on work was incremental with increased severity of lymphedema (range 22–75 %). The annual number of days off work for subclinical/mild lymphedema participants was 1.4 versus 8.1 days for moderate or severe participants (p = 0.003).

**Conclusions:**

This study identifies an additional detrimental effect of lymphedema on women’s work and career over and above the initial impact of breast cancer and provides empirical evidence for future prospective studies and policy improvement.

## Background

As the number of breast cancer survivors increases with better treatments, the number of patients with long-term side-effects including fatigue, cognitive problems, sexual dysfunction and fear of recurrence is also growing (Beckjord et al. 2014). Another feared side-effect is lymphedema caused by surgery, radiation therapy, and some chemotherapy treatments that increase the risk of fluid accumulation from lymphatic disruption (Cornish et al. 2000; Kilbreath et al. 2013). Lymphedema can cause pain, increase the risk of cellulitis, and limit a patient’s activities of daily living including, bathing, dressing, grooming and domestic tasks (Tretbar et al. 2008).

Lymphedema may present immediately or many years after breast cancer treatment. The mean interval from treatment to the development of mild arm lymphedema is about 18 months with one in three patients progressing from mild to severe arm lymphedema within 5 years (Bar et al. 2010, 2012). The incidence of breast cancer related lymphedema (BCRL) is variable and often under-reported due to a lack of standardised diagnostic criteria (Armer et al. 2013; Bernas 2013; Sander et al. 2002). However, recent studies have demonstrated that rates range from 5 % with conservative treatment (lumpectomy or wide local excision and sentinel node biopsy) alone, to greater than 20–50 % in cases with axillary node dissections, regional irradiation, and possibly taxane based chemotherapy (Hayes et al. 2005; Hayes 2008; Lucci et al. 2007; DiSipio et al. 2013; Swaroop et al. 2015). Age, obesity, nodal radiation, a post-operative seroma or infection further increase the risk (Monleon et al. 2015; Shaitelman et al. 2015).

Previous studies have examined the impact of cancer treatment on work and most of these have been on breast cancer. In a 2011 meta-analysis, 28 of 64 studies reported data about rates of employment or return to work after treatment. Overall, an average 63.5 % of participants (range 24–94 %) managed to return to work but the rate steadily increased as the period of time after cancer treatment increased. This ranged from, on an average, 40 % at 6 months post diagnosis to 62 % at 12 months, 73 % at 18 months, and to 89 % at 24 months after cancer diagnosis (Mehnert 2011).

While the literature that examines the impact of lymphedema on individuals’ employment is limited, it does report some consistent findings (Bulley et al. 2013; Gartner et al. 2010; Johansson et al. 2003; Fu et al. 2008). A common theme concerns whether the individual remains in employment as well as how many hours they choose to work. Exiting the workforce or reducing hours may occur for a variety of reasons, such as: pain and restricted arm mobility affecting the ability to complete tasks; infections causing absences; restriction on the wearing of compression sleeves or gloves in specific occupations and reduced mental health, worry about job security due to inability to accomplish assigned responsibilities, depression especially when one’s job responsibilities are impacted and feeling helpless due to loss of independence by having to rely on others to accomplish house work or job responsibility (Fu et al. 2013) (REF).

To that end, Bulley et al. (2013) examined the physical and psychosocial burden associated with lymphedema, noting that participants with lymphedema experienced greater burden than those without lymphedema with a doubling in the rate of stopping work or reducing hours. The importance of employment has also been highlighted in medical literature examining individuals’ Health Related Quality of Life (HR-QOL) with scholars emphasising the adjustments individuals have had to make to return to work post-diagnosis, such as changing employers, reducing hours or modifying their work space to accommodate their aching limbs (Fong et al. 2015).

No previous study has specifically explored the impact of the severity of lymphedema on work and career. With this in mind we undertook a cross-sectional quantitative study to further the scholarship on the impact of lymphedema over and above breast cancer with regard to work and career.

## Methods

### Setting

A survey of breast cancer survivors with and without lymphedema was undertaken Australia-wide. Participants were asked to complete an electronic survey examining the impact of lymphedema over and above breast cancer on their work, social life, self-esteem, body image and finances.

### Study population

Due to the limited knowledge on the socio-economic impact of lymphedema, an exploratory qualitative study was initially undertaken, which entailed interviews with 30 individuals—10 with primary lymphedema and 20 with secondary lymphedema. In those interviews, we explored two domains: employment and home-life. In addition, interviewees were asked to explain the treatment costs they have had to pay for over the course of their condition and how these affected their decision-making processes regarding treatment. This stage, to be reported elsewhere, allowed us to refine our conceptual framework and theory to test in the second, quantitative phase, reported here.

During the second phase, we utilized survey-methodology to collect extensive data on the impact that living with secondary lymphedema has on cancer-survivors’ work life. The survey instrument is available on request. A complexity that was addressed in the study was how to differentiate the impact of a diagnosis of lymphedema over and above a diagnosis of breast cancer. The survey instrument therefore had two sections looking at the impact of lymphedema first (if present) and then breast cancer for all patients.

Individuals eligible for participation were: female; over 18 years of age; previously diagnosed with primary stage I, II or III breast cancer who had completed treatment at least 1 year prior to recruitment and fluent in English. Individuals who fulfilled these criteria only became the control group. In addition, we targeted individuals who fulfilled all the criteria above, but with a confirmed diagnosis of lymphedema, either by a doctor or lymphedema therapist, including participants with subclinical lymphedema diagnosed with bioimpedance spectroscopy (L-Dex) alone; who had sought therapist advice; and/or were wearing compression garments. Participants completed the study questionnaire online.

Women previously diagnosed with breast cancer were approached for study participation through an Australian community-based breast cancer consumer organization, the Breast Cancer Network of Australia (BCNA). An e-mail invitation was sent by a contact person within the BCNA to members who had previously agreed to receive notifications about research studies. Participants with lymphedema were also asked to consider the study through the Australasian Lymphology Association (ALA) and by notices in the clinics of authors (JB, LK and HM). It was the responsibility of the women who received the e-mail to determine their eligibility for the study. A total of 361 women agreed to participate. Following online consent, participants anonymously completed the questionnaire that took approximately 30 min to complete. The conduct of this research was approved by the Macquarie University Human Research Ethics Committee.

### Definitions

We asked a screening question in order to classify our respondents’ lymphoedema stage. We asked them to reflect on their condition for the last month and first, report on its severity by choosing one of the following categories.

- No problem: no noticeable swelling. We later termed this category as sub-clinical lymphedema detected by a therapist or clinician using girth measures or bioimpedance spectroscopy (L-Dex).
- Mild lymphedema: soft swelling that is not obvious to others and comes and goes.
- Moderate lymphedema: swelling with occasional hardness in some areas that is obvious to others and is always present.
- Severe lymphedema: profuse swelling with thickened skin, constant hardness, and a very large, heavy arm that is extremely obvious to others and is always present.

### Statistical analysis

Participants with breast cancer were asked specific questions about how their cancer affected the following domains: (1) Work/career; (2) Family Life (3) Social/Leisure (4) Self Image and (5) Feeling about Self. For participants given a diagnosis of lymphedema, in addition to the above domains, data was also collected on the impact of lymphedema on employment, cost of seeing therapists and the cost of compression sleeves. Data collection occurred between November 2014 and March 2015 using Qualtrics. All *p* values are two-sided using the two-sample t test, unless otherwise specified. This paper will focus on the impact of lymphedema over and above breast cancer on work and career.

## Results

Of 361 participants, 209 (58 %) had breast cancer (BC) (Group 1) and 152 (42 %) had a diagnosis of BC and lymphedema (BC + LE) (Group 2). The severity of lymphedema was “not noticeable” in 14 (9 %), mild in 77 (51 %), moderate in 55 (36 %) and severe in six participants (4 %). Ninety-two of 209 (44 %) BC participants were aged under 55 compared to 54 of 152 (34 %) of BC + LE participants (p = 0.105). The duration since completion of all breast cancer treatment was <5 years for 75 % of the BC group and 56 % for the BC + LE group (p < 0.001). The time since the onset of lymphedema was <5 years in 65 % of the BC + LE group. Other demographic features of the study participants are shown in Table 1.

**Table 1:** Demographics of participant group

	Breast cancer	Breast cancer and lymphedema	Pearson Chi square p value
	209 (%)	152 (%)	
Age at time of survey			
<55 years	44.0	35.5	NS
≥55 years	56.0	64.5	
Country of birth			
Australia	80.4	80.3	NS
United Kingdom	9.6	8.6	
New Zealand	4.8	4.6	
Other	5.2	6.5	
Marital status			
Single, never married	9.1	6.6	NS
Married, de facto	75.6	80.3	
Separated/divorced	12.9	11.8	
Widowed	2.4	1.3	
Primary carer			
No	65.6	57.9	NS
Yes	18.2	22.4	
Yes	5.7	3.3	
Yes	3.3	5.9	
Yes	7.2	10.5	
Years since treatment of breast cancer			
<5 years	74.6	55.9	0.000
≥5 years	25.4	44.1	
Years since diagnosis of lymphedema			
<5 years	–	65.1	–
≥5 years		34.9	
Paid employment at diagnosis of breast cancer	77	63	0.004
Work industry			
Manufacturing	2.5	0.7	NS
Wholesaling	2.0	–	
Retailing	8.1	9.6	
Accommodation	1.0	1.4	
Cafés, restaurants	1.5	1.4	
House construction	0.5	1.4	
Health service	20.7	15.1	
Education	22.7	27.4	
Community care service	4.5	5.5	
Telecommunication	0.5	1.4	
Financial services	3.0	6.8	
Other	32.8	29.5	
Total household income			
≤$45,000	20.6	15.1	NS
>$45,000–<$100,000	29.2	31.6	
≥100,000	32.1	34.9	
Prefer not to say	18.2	18.4	

*$* Australian dollars, *NS* not significant

**Fig. 1:**
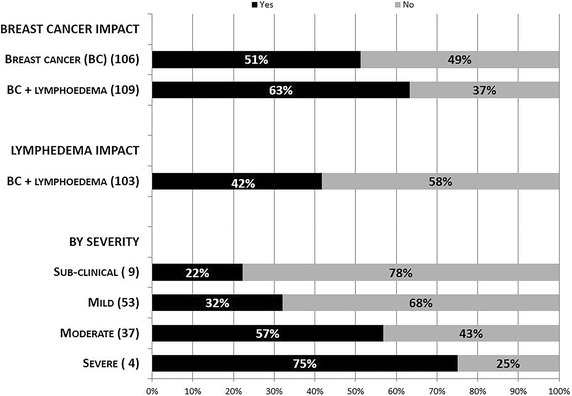
The relationship between breast cancer, lymphedema and lymphedema severity on their impact on the ability to work. Numbers in *parentheses* represent the total number of participants who were in paid employment within the various subgroups

Both breast cancer and lymphedema had a significant impact on a person’s ability to work. Breast cancer had an impact on the ability to work in 51 % of participants in Group 1 but participants with lymphedema perceived their breast cancer diagnosis to have had a greater impact on their work (63 %) (p = 0.03) (Fig. 1). Of the 103 Group 2 participants who were in paid employment at some time (either at diagnosis of lymphedema or at the time of the survey), 43 (42 %) indicated that lymphedema impacted their work performance. The impact of lymphedema on work increased as the severity of the condition increased, ranging from 22 % for subclinical lymphedema to 75 % for participants with severe lymphedema. The average time off work annually as sick or unpaid leave was less than 2 days (range 0–28 days) for subclinical or mild lymphedema (n = 50) and 8 days (range 0–54) for moderate or severe lymphedema (n = 28) (p = 0.003).

**Fig. 2:**
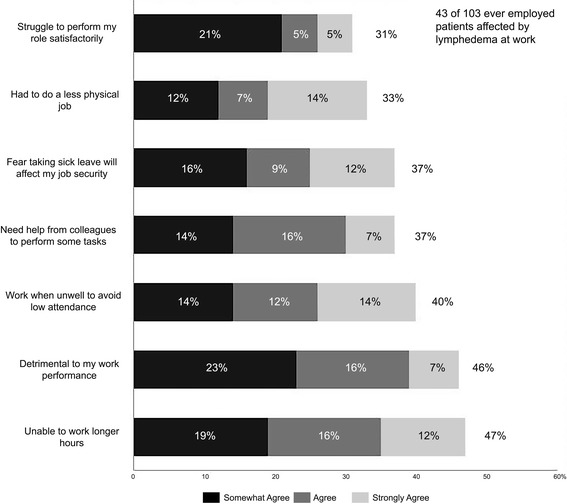
Reasons why employed participants with lymphoedema were affected at work. Percentages with *parentheses* are of the 103 total and without *parentheses* are of the 43 participants whose diagnosis of lymphedema affected them at work

Figure 2 explores some of the reasons and the extent to which work performance was affected in Group 2. Of the 43 participants, 40 % reported attending work when they were unwell for fear of losing their job (a phenomenon which Aronsson et al. (2000) termed “presenteeism”) and 47 % reported not being able to work longer hours. These numbers correspond to 17 and 20 % of the 103 participants who were employed.

Table 2 shows employment transitions for the two study groups. At the point of diagnosis, 77 % of participants in the Group 1 were in paid employment dropping to 59 % at the time of the survey (p = 0.025). For Group 2 the numbers drop from 63 to 51 % respectively (p = 0.165). The main reason behind this transition was an increased percentage of retirements (Group 1: 27 % vs Group 2: 37 %, p = 0.044).

**Table 2:** Transition between employment after diagnosis of breast cancer or breast cancer and lymphedema

	Breast cancer (BC)		Breast cancer + lymphedema	
	209		152	
	When first diagnosed (%)	Now (%)	When first diagnosed (%)	Now (%)
*Paid employment*	77	59	63	51
Yes, full-time	38	23	34	22
Yes, part-time	22	22	19	21
Yes, casual	7	8	3	2
Yes, self-employed	10	6	7	6
*Not employed*	22	41	37	49
No, looking for a job	0	3	1	3
No, retired	12	27	21	37
No, other	10	11	15	9
Total	100	100	100	100

We explored how often and why employment changed after a diagnosis of breast cancer or lymphedema (Table 3). Of the participants who were employed in Group 1, just over half (51 %) indicated that their conditions of employment changed mostly due to reduced working hours. At the time of diagnosis of lymphedema, about one in five (19 %) in Group 2 indicated that their employment conditions had changed again mainly due to reduced hours.

**Table 3:** Transition between employment after diagnosis of breast cancer or lymphedema

	Employment changes due to breast cancer		Employment changes due to lymphedema	
	166		103	
	No.	%	No.	%
Yes, employment changed	84	51	20	19
Reduced working hours	39	23	10	10
Stopped working	27	16	5	5
Changed role	21	13	4	4
Changed employer	15	9	1	1
Self-employed now	3	2	2	2

Only includes participants who indicated that the diagnosis of breast cancer or lymphedema affected their employment and multiple reasons could apply to each patient

Of the 103 Group 2 participants who were in paid employment, 54 (52.4 %) did not disclose their diagnosis of lymphedema to their supervisor and 40 (38.8 %) did not disclose their diagnosis of lymphoedema to their coworkers (p = 0.147). Table 4 shows the participants’ perceptions of how their peers and coworkers reacted to their diagnosis of lymphedema. Although there was a lower rate of disclosure to supervisors (47.6 %) than peers (61.2 %), once disclosed there was no significant difference in the type of information that was disclosed. Although the rate of discrimination at work was thought to be higher in the BC + LE group (14 %) than the breast cancer group (5 %), this did not reach statistical significance (0.07).

**Table 4:** Reaction of supervisors and co-workers on diagnosis of lymphedema

No. participants employed (103)	Told co-workers (n = 63; 61.2 %)						Told supervisor (n = 49; 47.6 %)					
	Mean	SD	Somewhat agree (%)	Agree (%)	Strongly agree (%)	% Agreement	Mean	SD	Somewhat agree (%)	Agree (%)	Strongly agree (%)	% Agreement
They treated me as usual	6.05	0.99	11	49	33	93	5.84	1.21	10	49	29	88
They demonstrated understanding	5.11	1.54	16	37	16	68	5.10	1.58	20	45	10	76
They were generally supportive	5.75	1.09	13	48	24	84	5.53	1.44	12	53	18	84
They provided practical support-e.g. help lifting	4.56	1.92	13	24	17	54	4.33	1.94	8	27	12	47
They were not concerned about my condition	3.63	1.78	14	19	2	35	3.57	1.90	14	14	6	35
They were too busy to pay much attention	3.52	1.77	11	14	3	29	3.35	1.83	16	10	4	31
They did not trust my capabilities	2.41	1.44	5	3	0	8	2.39	1.58	4	0	4	8
They thought I used my condition as an excuse	1.95	1.33	5	2	0	6	2.20	1.68	10	2	2	14
I have experienced discrimination	1.94	1.37	2	2	2	5	2.31	1.79	8	0	6	14
I have been excluded from career-advancement opportunities	2.05	1.52	3	3	2	8	2.43	1.88	10	2	6	18

## Discussion

Lymphedema is a feared complication of breast cancer and impacts physical, functional, psychological and social well-being of participants after breast cancer treatment. Yet, existing scholarship is in early stages of development regarding many aspects of this condition. Our first in-depth cross-sectional study, shows that, when compared to breast cancer survivors without lymphedema, individuals living with lymphoedema are worse off in terms of work and career.

In a previous meta-analysis of the impact of a diagnosis of cancer on work, a non-supportive work environment, manual work, cancer types associated with an unfavourable prognosis, the presence of fatigue and physical symptoms, and perceived employer discrimination because of cancer and treatment were reported as barriers for returning to work (Mehnert 2011). Our study highlights that sick leave, in its current form, is falling short for individuals living with chronic illnesses: 43 of 103 employed participants (42 %) reported that lymphedema had affected their work performance with 17 % of the total reporting that they have turned up at work on days they feel unwell either to avoid a low attendance record, or because they fear for their job security (Fig. 1).

Few studies, however have examined the impact of lymphedema over and above the diagnosis of breast cancer. For example, Johansson et al. (2003) explored twelve working women’s experiences of lymphedema and reported that typing or long periods without rest became difficult or even impossible. Fu et al. (2008) examined the impact of lymphedema in the workplace among five female breast-cancer survivors and found that it was a particular problem for women who needed to lift objects at work. Fears regarding job security were also reported especially in cases where the employer was unsupportive. In an updated study Fu (2008) found that the majority of women (12 out 22) whose jobs involved heavy lifting and constant use of the affected arm and hand were from either African American or Chinese American groups. It was noted that this group of women needed their jobs as a source of financial income or medical insurance and not only suffered the physical and functional impact of lymphoedema on their work, but had to endure constant emotional distress created by their supervisors or employers who had no understanding of breast cancer survivors with lymphoedema. Based on these findings we argue that policy makers should pay further attention to the fact that sick leave is designed under an acute illness framework, which assumes the individual will eventually get better.

In a study of 67 participants with perceived lymphedema who were working, Mehnert (2011) found that 25 % had to stop their employment and 10 % (total 35 %) had to reduce their hours compared to 11 and 8 % of 247 participants (total 19 %) without lymphedema. Their study was similar to ours except they recruited from a specific follow-up clinic rather than a nationwide survey performed in our setting. In our study, of the 109 participants with BC + LE, 22 (20 %) had to stop working, 24 % had to reduce hours because of their breast cancer (total, 44 %), compared to 16 and 23 % of the 166 participants with BC alone (total, 39 %).

Gartner et al. (2010) also examined the impact of lymphedema on women’s daily activities at work, with 36 % of the sample indicating that it had affected their work. Specifically, 47 % reported light work above shoulder level as problematic, 27 % reported daily activity with involvement of shoulder rotation as troublesome while heavy work was associated with difficulties for 1884 women (59 %).

Finally, Fantoni et al. (2010) studied 379 women with breast cancer aged up to 60 years old, who were working at the time of diagnosis using a 45-item questionnaire. During a median follow-up of 36 months, 82.1 % of the 379 women who had worked before their diagnosis returned to work after a median sick leave of 10.8 months. Older age, lower educational level, chemotherapy, radiotherapy, lymphoedema, psychological or organizational self-perceived constraints related to their former job, and the lack of moral support from work colleagues both limited and delayed return to work.

Our study has limitations particularly as it used a cross-sectional rather than a longitudinal survey design. In addition, as we asked participants to self-report on the impact of lymphedema over and above breast cancer, the study could be characterised by recall bias. However, a cross-sectional design and the use of an online survey allowed for a good sample size, and indicators of lymphoedema status were included, such as number of symptoms, time since diagnosis and we included only participants who had seen a lymphedema therapist. We did not examine education level and subsequent impact on work but previous studies have found that cancer survivors were likely to be unemployed if they did not complete high-school, were previously receiving social security benefits and women were 23 % less likely to find a job after they received employment assistance and support, such as job-hunting services or on-the-job training than men (Chan et al. 2008).

These limitations notwithstanding, this is the largest study showing the impact of lymphedema over and above breast cancer, and shows how its impact worsens as the condition progresses. The issues are complicated and impact on work from an illness does depend on multiple competing factors. To adequately review these factors, we are planning a prospective study to further differentiate the impact on work of lymphedema versus the impact of breast cancer and its short-term treatments.

The findings from this study have implications for clinical practice, future research and for policy makers. Health professionals involved in the care of women with lymphedema need to be aware that these women are at risk of not only experiencing psychological distress and body image disturbance (Alcorso et al. 2016) but also additional detrimental effect of on women’s work and career, over and above the initial impact of breast cancer.
